# Approaching Sites of Action of Temozolomide for Pharmacological and Clinical Studies in Glioblastoma

**DOI:** 10.3390/biomedicines10010001

**Published:** 2021-12-21

**Authors:** Margaux Fresnais, Sevin Turcan, Dirk Theile, Johannes Ungermann, Yasmin Abou Zeed, Joshua Raoul Lindner, Marius Breitkopf, Jürgen Burhenne, Walter E. Haefeli, Rémi Longuespée

**Affiliations:** 1 Department of Clinical Pharmacology and Pharmacoepidemiology, Heidelberg University Hospital, Im Neuenheimer Feld 410, 69120 Heidelberg, Germany; Margaux.Fresnais@med.uni-heidelberg.de (M.F.); Dirk.Theile@med.uni-heidelberg.de (D.T.); johannes.ungermann@stud.uni-heidelberg.de (J.U.); Y.Abou@stud.uni-heidelberg.de (Y.A.Z.); joshua.lindner@stud.uni-heidelberg.de (J.R.L.); Breitkopf@stud.uni-heidelberg.de (M.B.); Juergen.Burhenne@med.uni-heidelberg.de (J.B.); Walter-Emil.Haefeli@med.uni-heidelberg.de (W.E.H.); 2 Neurology Clinic and National Center for Tumor Diseases, Heidelberg University Hospital, 69120 Heidelberg, Germany; Sevin.TurcanTaranda@med.uni-heidelberg.de

**Keywords:** temozolomide, glioblastoma, pharmacology, site of action, quantification, omics

## Abstract

Temozolomide (TMZ), together with bulk resection and focal radiotherapy, is currently a standard of care for glioblastoma. Absorption, distribution, metabolism, and excretion (ADME) parameters, together with the mode of action of TMZ, make its biochemical and biological action difficult to understand. Accurate understanding of the mode of action of TMZ and the monitoring of TMZ at its anatomical, cellular, and molecular sites of action (SOAs) would greatly benefit precision medicine and the development of novel therapeutic approaches in combination with TMZ. In the present perspective article, we summarize the known ADME parameters and modes of action of TMZ, and we review the possible methodological options to monitor TMZ at its SOAs. We focus our descriptions of methodologies on mass spectrometry-based approaches, and all related considerations are taken into account regarding the avoidance of artifacts in mass spectrometric analysis during sampling, sample preparation, and the evaluation of results. Finally, we provide an overview of potential applications for precision medicine and drug development.

## 1. Introduction

Glioblastoma (GBM) is an aggressive primary brain tumor in which the invasive growth pattern cannot be completely eliminated by tumor debulking surgery [1,2]. Administration of temozolomide (TMZ) to GBM patients, together with tumor debulking and focal radiotherapy, prolongs GBM patients’ survival from 12.1 to 14.6 months, compared to radiotherapy alone [3,4]. TMZ is a monofunctional alkylating imidazotetrazinone displaying bimolecular nucleophilic substitution (SN2 mechanism), with a simple methyl group transferred to the final molecular targets [5]. Understanding the actual biochemical action of TMZ could have a positive impact on precision medicine and drug development [6]. Indeed, new drugs for GBM (including targeted therapies) are often tested as comedications with TMZ. Finely monitoring the biochemical action of TMZ would also permit the monitoring of the drug-response relationship with new drugs more accurately. The metabolism of TMZ and its known action give some clues to the pathways which should be followed in order to monitor its effect at its anatomical, cellular, and molecular sites of action (SOAs) [6]. In the present manuscript, we refer to the general parameters to be evaluated when studying drugs at their SOAs, as described previously [6]. These parameters, as specified and described in [6], are given in Table 1.

Mass spectrometric methods such as liquid chromatography–tandem mass spectrometry (LC-MS/MS) and desorption/ionization (DI) methods such as matrix-assisted laser desorption ionization (MALDI) are commonly used to quantify drugs at their SOA [6]. LC-MS/MS, as a standalone approach, is currently the gold standard for the quantification of drugs in biological matrices [7]. Results from LC-MS/MS also provide strong support for the development of alternative MS-based approaches, e.g., on-surface imaging and/or profiling using MALDI-MS. In this perspective article, we focus on LC-MS/MS and MALDI-based approaches, as well as their interplay, to detect TMZ and its effects at its SOAs, as well as analytical workflows suitable for this purpose.

## 2. Understanding and Monitoring Temozolomide Fate and Biochemical Action

### 2.1. Absorption, Distribution, Metabolism, and Excretion of Temozolomide

**Figure 1:**
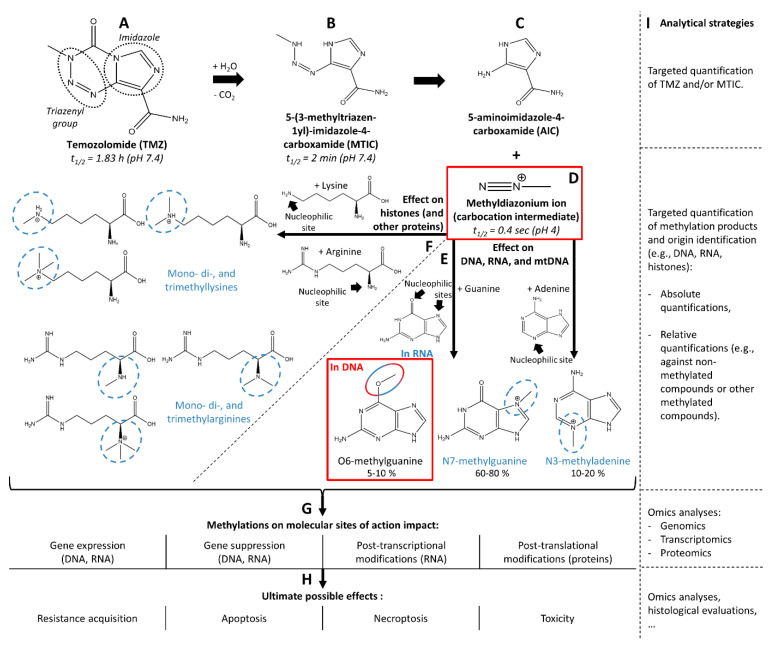
Chemical decay of temozolomide (TMZ, **A**) into 5-(3-methyltriazen-1-yl)-imidazole-4-carboxamide (MTIC, **B**), 5-aminoimidazole-4-carboxamide (AIC, **C**) and the carbocation intermediate, methyldiazonium ion (**D**). The methyldiazonium ion further reacts with guanine and adenine from DNA, mitochondrial DNA (mtDNA), and RNA to form O6-methylguanine, N7-methylguanine, and N3-methyladenine (**E**). Alternatively, the methyldiazonium ion also reacts with lysine and arginine from histones, and possibly other proteins, to give mono-, di-, and trimethylated forms of lysine and arginine (**F**). Active TMZ metabolite and active methylated compounds from the intended molecular site of action (SOA) are highlighted in red, while methylated forms from alternative molecular SOAs are highlighted in blue. The potential internal modifications and ultimate biological effects of the methylation at both intended and alternative sites of action are given in (**G**,**H**), respectively, and the possible analytical strategies to monitor the fate and action of TMZ are listed in (**I**).

TMZ (trade name Temodal or Temodar, Figure 1A) is prescribed in tablets and is absorbed in the digestive tract. TMZ is stable at low pH in the stomach, but is labile at physiological pH (i.e., 7.4) during its absorption in the gut and its further distribution in plasma, where it has a half-life of 1.8 h [8] and spontaneously hydrolyses to 5-(3-methyltriazen-1yl)-imidazole-4-carboxamide (MTIC, Figure 1B) [8]. MTIC has a half-life of 2 min and decomposes into 5-aminoimidazole-4-carboxamide (AIC, Figure 1C) and methyldiazonium ion (Figure 1D), which is the highly electrophilic final carbocation intermediate and which reacts immediately with potential molecular targets and has a half-life of only 0.4 s [5]. TMZ crosses the blood-brain barrier (BBB) and is found in the brain [5,9,10,11]. However, the short half-life of TMZ, together with issues related to permeability of the BBB and blood circulation in the brain tumor [12], could explain the fact that only 20% of the plasma concentration (Table 1, P2) reaches the brain tissue (Table 1, P5), as demonstrated using microdialysis [11]. The metabolites of TMZ have half-lives that are too short to be traceable (Table 1, P9), but simulations using admetSAR, a tool for the prediction and optimization of chemical ADME and toxicity properties [13], suggest that MTIC should also, in principle, cross the BBB [5]. Although TMZ is a prodrug, it is spontaneously hydrolyzed, and no metabolic enzyme is involved in its processing (Table 1, P10). Both TMZ and MTIC display a T_max_ at 1.5 h in plasma [14], thus indicating that besides its half-life of 1.8 h, TMZ decays during adsorption. As well as reaching its aimed anatomical SOA (brain), TMZ and its metabolites reach different anatomical sites and various cell types for further (off-target) methylation of various classes of biomolecules (Table 1, P8). Changing dose (Table 1, P1), frequency, and delivery method to improve BBB penetration does not improve patient outcomes but results in higher toxicity [15], suggesting that off-target effects are responsible for toxicity. The chemical fate of TMZ is illustrated in Figure 1A–F.

### 2.2. Chemical Action of Temozolomide

The final carbocation metabolite methyldiazonium ion (Figure 1D) is a highly electrophilic compound, which reacts with nucleophilic moieties of a large panel of biomolecules. Intuitively, methyldiazonium reacts with the more nucleophilic moieties. The molecular SOAs are the purine bases of DNA, guanine, and adenine. Most methylations in DNA (60–80%) take place at position N7 of guanine, which is the most nucleophilic moiety of all purines [16,17]. Approximately 5–10% of the methylations take place at position O6 of guanine, and the remaining 10–20% at position N3 of adenines [16,17]. These chemical events are illustrated in Figure 1E.

### 2.3. Biological Action of Temozolomide

**Figure 2:**
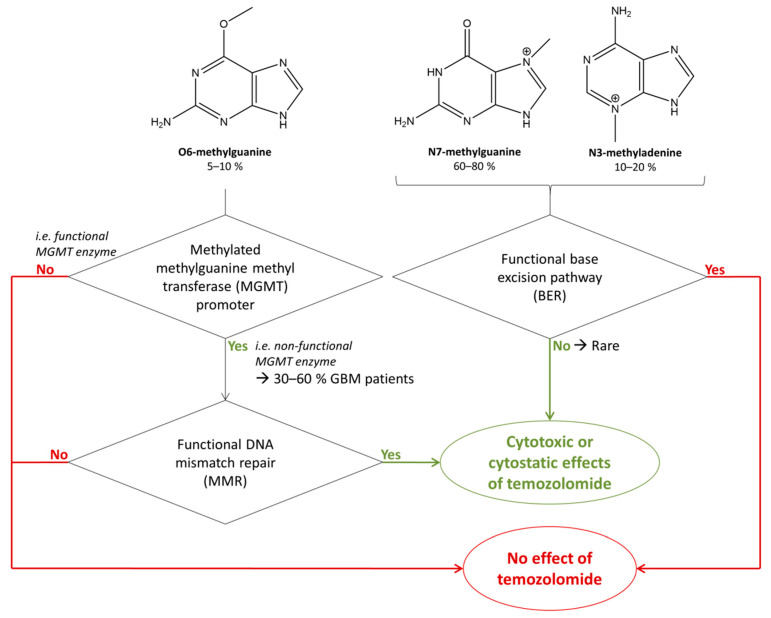
Biochemical parameters allowing the action of temozolomide (TMZ) through its metabolites O6-methylguanine, N7-methylguanine, and N3-methyladenine. Pathways leading to an action of TMZ are shown in green and pathways opposing its action leading to drug resistance in red.

The downstream action of guanine methylation depends either on efficient or deficient DNA repair machineries, as illustrated in Figure 2. Efficient base excision repair (BER) machinery repairs DNA with incorporated N3-methyladenine or N7-methylguanine moieties and leads to drug resistance [17]. A non-functional BER would induce a response to TMZ due to N3-methyladenine or N7-methylguanine, but BER is rarely inactivated in GBM [17]. Consequently, although N7-methylguanine and N3-methyladenine are the most common methylation products in DNA, they rarely affect the cell [5]. Methylations of O6-methylguanines are primarily repaired with a functional suicide enzyme methylguanine methyl transferase (MGMT) [17,18], which leads to treatment resistance. However, in 30–60% of GBM patients, this enzyme is not functional, due to the methylations of its promoter [19,20,21]. In that regard, the stratification of low-grade glioma patients based on isocitrate dehydrogenase (IDH) mutation status is relevant to the prediction of their clinical response to TMZ [22,23]. IDH mutation is related to epigenetic changes such as CpG island hypermethylations [22], which can take place in the promoter of MGMT, thus silencing its expression and functioning [24]. Therefore, IDH mutation is correlated with MGMT promoter methylation and a higher rate of response to TMZ [24,25]. In the absence of methylation of the MGMT promoter, the MGMT enzyme is then functional and so repairs the methylation of O6-methylguanines, leading to little or no benefit for TMZ treatment [25]. For these reasons, the methylation of the MGMT promoter is in the process of becoming a major predictive marker for guiding the decision to dose GBM patients with TMZ [25].

In the presence of a non-functional MGMT, O6-methylguanine can be replaced by thymine, and functional mismatch repair (MMR) machinery leads to efficient action of TMZ, with either DNA double strand breaks (causing autophagy) or futile cycling (promoting cell death) [17,18]. Therefore, although the percentage of methylation of guanines at position O6 is quantitatively low compared to the other methylations, the biological action of TMZ mainly relies on this modification and the association of dysfunctional MGMT and functional MMR machineries.

In terms of ultimate biological effects on cells, cytostatic models are currently preferred to cytotoxic ones [26,27].

It is important to note that the chemical reaction of the electrophilic methyldiazonium ion is not specific to purine bases but can occur with many other nucleophilic moieties. Known chemical side effects of methyldiazonium include the methylation of RNA [28,29], mitochondrial DNA (mtDNA), the ribose-phosphodiester backbone of DNA, and other macromolecules such as proteins [5]. Methylations of RNA and mtDNA are important to consider for the design of analytical workflows because they can create important artifacts in the MS-based quantification of the intended modified molecular SOAs (Figure 1E, see Section 3.2.2, “Retrieval of Deoxyribonucleosides”); but they may also be precious biological material in the study of the modified SOAs that may play a role in TMZ effects (Figure 1G,H). On the other hand, protein methylation (Figure 1F) can contribute to the overall anti-proliferative effect, but this is not detected if only DNA methylation is quantified. Methylation affects proteins of key signaling cascades such as the mitogen-activated protein kinase (MAPK), the Janus kinase (JAK), or the signal transducer and activator of transcription (STAT) pathways [30,31]. In addition, it has been proven that TMZ can also lead to histone alkylation and can possibly act through the epigenetic regulation of protein [32]. Based on this knowledge, it is important to consider two types of SOAs for TMZ: intended SOAs (DNA in brain tumor cells) and alternative SOAs (e.g., mtDNA, RNA, and proteins in the brain and other tissues), leading to intended effects of TMZ (i.e., cytotoxicity or cell arrest of the tumor cell, tumor reduction) or to side-effects (i.e., cytotoxicity or cell arrest of other cells and overall toxicity). It is likely that the beneficial and adverse effects of TMZ are due to a combination of actions at intended and alternative SOAs. Therefore, comprehensive investigations of the action of TMZ would necessitate the consideration of all possible molecular interactors that may play a role in the eventual expected and unexpected beneficial or adverse effects of TMZ.

All these elements give hints for the route to follow to monitor the action of TMZ on GBM, in terms of the quantification of the chemical action of TMZ and the evaluation of its biological effects. In the present perspective article, we emphasize the study of the intended SOAs of TMZ and provide insights on different options for studying alternative SOAs (Figure 1I).

### 2.4. Retrieval of the Intended Anatomical Site of Action of Temozolomide

Determining how to study the action of TMZ at its SOAs in human brain tumors requires having a clear knowledge of treatment and tissue collection timelines. Treatment of GBMs includes maximal surgical resection, followed by radiotherapy and concomitant oral TMZ (75 mg/m^2^) chemotherapy, followed by adjuvant chemotherapy with TMZ at a dose of 150 to 200 mg/m^2^ for five days every 28 days over six months. The addition of TMZ to radiation (the ‘Stupp protocol’) extends survival by a median of 2.5 months compared to radiation alone. However, despite multi-modal treatment, tumor progression occurs in the majority of patients, and the median survival is less than 15 months. The treatment course for patients with recurrent GBM depends on several factors, including age, prior therapy, MGMT promoter methylation, and disease progression patterns [33]. Overall, less than 50% of patients are eligible for a second surgery when tumor relapse occurs. The second surgery is feasible if recurrence is > six months after initial surgery, depending on clinical parameters and tumor characteristics, and it should provide a benefit to the patient [33,34].

It is important to note that the timeline for tissue collection in patients (biopsies) during clinical trials differs from the timeline for surgery after treatment and depends on the trial. Depending on the drug used in combination with TMZ, biopsies can be performed at closer time points after treatment.

The extent of a study on TMZ in treated brain tumors depends, therefore, on the heterogeneous timelines of surgery and treatment between patients and on the patient-dependent effects of TMZ. The fate of damaged tumor cells may vary (resistance, necroptosis, apoptosis) and take place, more or less, in the short term after treatment. The study of the effects of TMZ is possible if such damaged cells allow for the detection of the unbound fraction of TMZ, methylated guanines, and alternative SOAs. Alternatively, alkylated purine may remain in the normal brain tissue microenvironment after cell death and decay. In any of these events, the quantification of alkylated guanines may be possible if the surgery takes place when damaged cells or remaining alkylated guanines are still present.

## 3. Analytical Workflows to Quantify the Intended Chemical Action of Temozolomide in Glioblastoma

**Figure 3:**
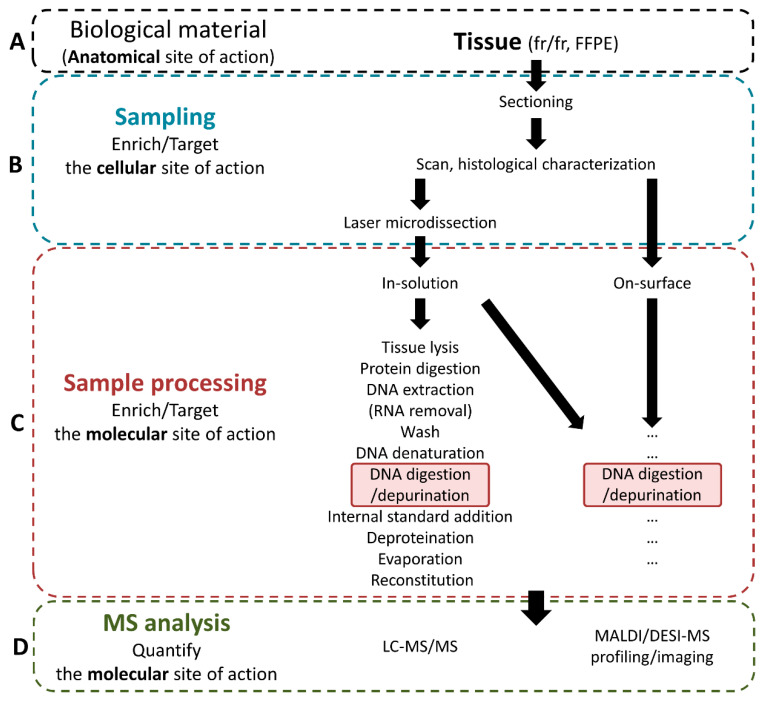
Design of the analytical workflow to monitor temozolomide (TMZ) at its intended anatomical, cellular, and molecular sites of action (SOAs). Fresh frozen (fr/fr) or formalin-fixed and paraffin-embedded (FFPE) tissue from TMZ anatomical SOA (**A**) is further sampled to enrich the cellular SOA (**B**) and processed following in-solution or on-surface workflows (**C**, a selected generic workflow inspired by [42] is displayed with optional steps in brackets) to monitor the effects at the intended molecular SOA (i.e., by quantifying O6-methylguanines from cancer cell DNA) using mass spectrometry (MS) analysis by liquid chromatography (LC)-MS, matrix-assisted laser desorption ionization (MALDI)-MS, or desorption electrospray ionization (DESI)-MS (**D**). “…“ refers to additional sample processing steps that may be adapted to the procedure for on-surface analysis if relevant.

As detailed above, to monitor the action of TMZ in GBM, the analytical approaches used to process and analyze the samples retrieved from the anatomical SOA (i.e., tumor tissue, Figure 3A) should first focus on targeting the intended cellular and molecular SOAs (i.e., cancer cells, and O6-position on DNA guanines, respectively). In addition, it might be relevant to monitor other cellular and molecular information, such as the levels of native guanines and adenines, and the levels of the other methylated forms of these purines, on DNA, but also on RNA. Depending on the targeted molecular information, the analytical workflow will need to be adapted from the usual workflows as in Figure 3.

As commonly applied for MS-based DNA/RNA analyses of tissue samples, three main steps have to be considered when conceptualizing a suitable workflow to monitor TMZ action at the anatomical SOA: (i) tissue sampling, (ii) in-solution (LC-MS/MS) or on-surface (MALDI-MS) sample processing, and (iii) respective analysis (Figure 3). Each step influences the subsequent step, and every workflow has to be considered comprehensively. Below, we describe the most promising parameters and procedures to quantify the action of TMZ at its SOAs.

### 3.1. Sampling of the Intended Cellular Site of Action

Depending on the established protocol of sample collection during a surgical procedure, the starting tissue material (Figure 3A) may be fresh/frozen (fr/fr) or formalin-fixed and paraffin-embedded (FFPE). In the context of the personalized monitoring of the action of TMZ, it is important to consider that biopsies would be obtained during surgery, which would usually provide tissue pieces of few mm^3^. The choice of the sampling method (Figure 3B) should then be adapted to low-volume samples. In this context, tissue sections are the most versatile samples, allowing histological examination, sample processing, and analysis from very limited amounts/volumes. GBM tumors can be very diffuse and tumor cell content can be limited and heterogeneous between specimens and within a single tissue specimen. Serial tissue sections can be performed and further processed for histological staining (e.g., hematoxylin and eosin (HE)) and scanned in high resolution for further histological examination and localization of the cellular SOA of interest (Table 1, P3). In this regard, digital pathology plays a considerable role in histological visualization and cellular recognition for further cell type classification and counting [35,36,37,38,39]. Cell characterization is a preprocessing step that represents a major asset for aspects of quantification. It allows precise control of the cell types and adjustment for their number in a sample for further in-solution processing. When using an on-surface quantification method, the type and number of cells per area can be monitored (see Section 3.2. Ultimately, the abundance of methylated guanines will likely depend on their local availability, the activity of repair mechanisms, and on the fate of damaged tumor cells (necroptosis, apoptosis). Therefore, it would be of utmost importance to identify the nature of the cells to be collected before sample processing. In that context, the above-mentioned tools for digital pathology would permit the full control of cell contents to be handled and avoid any artifacts due to sample heterogeneity when performing sample comparisons. It may also be possible that alkylated DNA also remains in the brain after cell death if cytotoxicity occurs. In this case, even in the absence of damaged cells, brain tissue would have to be considered for the quantification of alkylated purines.

If tissue sections serve as specimens for in-solution quantification, laser microdissection (LMD) allows for the controlled selection and isolation of cell types of interest before further sample processing [40]. Conversely, on-surface sample processing would be performed directly (i) from regions of interest (ROIs) in tissue sections when MALDI profiling is applied, or (ii) on the whole tissue section when MALDI-MSI is applied [7,41].

### 3.2. Sample Processing for Intended Sites of Action of Temozolomide

A brain tissue section may then be the most suitable material for further processing. Two options are possible: (i) in-solution and (ii) on-surface sample processing (Figure 3C).

In-solution processing usually requires tissue lysis/homogenization at certain conditions (e.g., solvent, temperature, pH, ultra-turrax, vortex, ultrasonication) in order to expose the unbound compound (i.e., TMZ) and metabolites, if applicable, for extraction and subsequent quantitative analysis with LC-MS/MS. For the quantitative analysis of native and methylated purines/nucleosides by LC-MS/MS, further digestion procedures under controlled conditions are required.

For on-surface analyses as well, approaches also differ for the analyses of the unbound or bound fractions of the compounds. Sample processing would also require different approaches depending on the final type of analysis: profiling or imaging. The deposition of reagents using profiling approaches can be performed by hand-pipetting or automatic micro-/nano-spotting.

Imaging methods necessitate the automatic deposition of reagents (e.g., using robotic sprayers or spotters) [7]. Both imaging and profiling methods require samples to be digested in incubation chambers with stable and homogeneous humidity [43] for the analysis of fractions of drugs covalently bound to their molecular SOA [6]. Any procedure involving washing steps may alter the histological structures in tissues, in particular, fr/fr tissues. In this context, correlation with histological information from serial tissue sections would not be possible; therefore, washing steps must be avoided.

Overall, in-solution processing might be more preferable than on-surface processing because this method may provide more efficient extraction/digestion yields of unbound TMZ or of O6-methylguanines, respectively, and would likely be more sensitive. However, in-solution processing without prior selection of a region of interest is unable to maintain the localization of unbound TMZ or of O6-methylguanines in tissue samples. In this context, when the sampling can be spatially controlled (e.g., using LMD), in-solution processing may be preferred for further LC-MS/MS analysis, whereas on-surface processing may be reserved for on-surface MALDI-MS or DESI-MS analyses (Figure 3D).

Although LMD allows for the controlled collection of cell types at specific numbers before in-solution processing, it further reduces sample volumes. Therefore, microprocessing may have to be considered for biochemical processing. Microprocessing workflows require special considerations in order to avoid sample loss; these considerations primarily involve reducing the number of procedure steps and avoiding sample transfer between tubes as much as possible [44]. Nevertheless, essential preparation steps must be maintained; in the case of TMZ, these are DNA and possibly protein digestion. Therefore, implementation of this method requires a thorough review of the biochemical steps that could be omitted while still allowing for optimal digestion/processing with minimized sample loss. For example, the difficulty of lysing a tissue depends on its origin or preprocessing (fr/fr or FFPE), and the absence of fixatives in fr/fr tissues favors their lysis, whereas lysis of FFPE tissue is less easy. However, our experience with microproteomics of FFPE tissues indicates that it is possible to digest proteins directly on the tissue piece, even without previous tissue lysis or protein extraction [41].

#### 3.2.1. Retrieval of Unbound Temozolomide at the Intended Anatomical Site of Action

To assess exposure-effect relationships and pharmacokinetic parameters, the unbound drug fraction is usually monitored in plasma (Table 1, P2), but the evaluation of unbound drug levels at the anatomical SOA (Table 1, P5) can also give precious information regarding exposure. As mentioned before, 20% of the unbound TMZ fraction in plasma crosses the BBB and accumulates in the brain at relevant therapeutic concentrations. The measurement of unbound TMZ in brain tissue, or more specifically in GBM tissue, provides information about its disposition at the anatomical SOA, but without giving direct information about its actual biochemical effectiveness. Using ultra-performance LC (UPLC)-MS/MS, Goldwirt and co-workers compared the quantification of TMZ in plasma and in mouse brains [45] and showed that TMZ rapidly crosses the BBB with a delay of peak concentrations of approximately 30 min. The sample preparation of tissues, summarized in Table 2**,** was performed at low pH to protect TMZ from further spontaneous conversion into MTIC.

Thus far, TMZ has not been measured in tissue sections using MALDI-MSI. LC-MS/MS and MALDI-MSI would be informative for a proper assessment of the timing of TMZ distribution in animal samples, since the time points for tissue collection might be fully controllable in the context of an animal experiment. However, it would not be immediately applicable to human samples. The half-life of TMZ of 1.83 h and the treatment/surgery schedule (see Section 2.4) imply that TMZ would no longer be present in human biopsies. Moreover, even if the time course of TMZ’s interaction with its molecular SOA (via its carbocation intermediate, Figure 1D) is not well defined, the resulting covalent methylation of purines is a stable chemical modification (Table 1, P7) that is visible as long as the exposed cell is alive and lacks appropriate repair mechanisms. Therefore, evaluating the effect of TMZ at its molecular SOA may appear to be the only option to evaluate the relationship between the administered TMZ dose and the resulting effect at its cellular SOA. For this reason, in the following sections, we specifically address alternative/complementary options, such as monitoring methylated guanines, to study the action of TMZ.

#### 3.2.2. Retrieval of the Intended Modified Molecular Site of Action: Methylated Guanines

**Figure 4:**
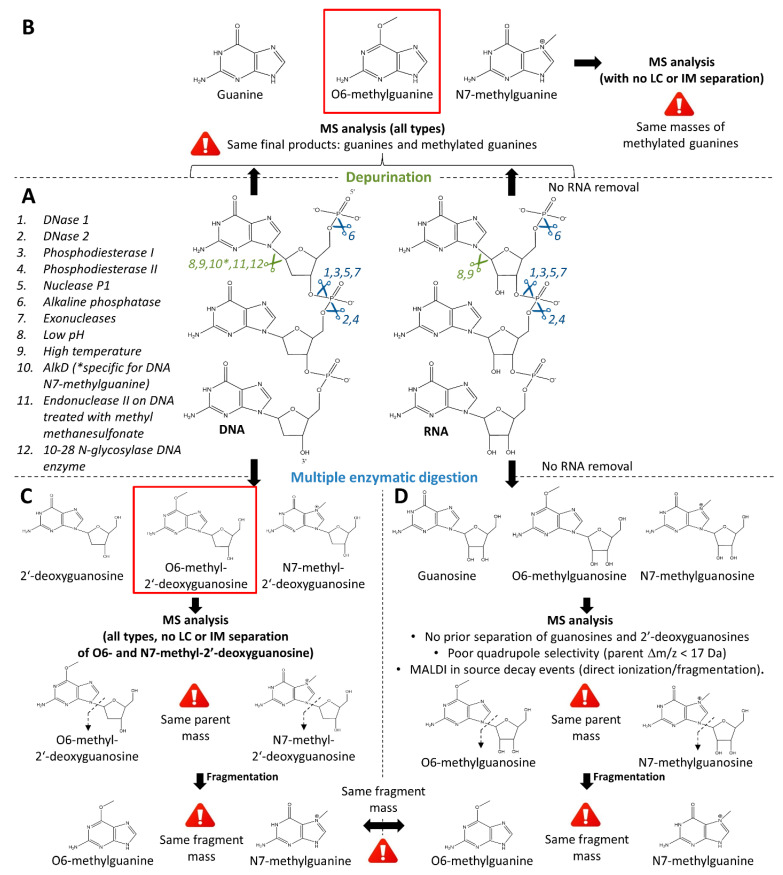
Chemical products of DNA and RNA depurination and enzymatic nucleoside digestion and possible analytical artifacts. Before MS-based analysis, one of the important steps is enzymatic digestion or depurination of DNA, as well as RNA, if no RNA removal step was performed (**A**). DNA and RNA depurination give the same final products, i.e., purines and methylated purines, regardless of their origin (**B**). DNA multiple enzymatic digestion yields 2′-deoxynucleosides and methyl-2′-deoxynucleosides, which are further fragmented in the MS device (**C**), and RNA multiple enzymatic digestion yields nucleosides and methylnucleosides, which are also further fragmented in the MS device (**D**) into the same fragments (nucleobases) as for DNA digestion. For each of these reactions, methylation at position O6 or N7 yields parent (methyl-2′-deoxyguanosines) and daughter (methylguanines) ions with the same mass. Main products of interest for the monitoring of TMZ action (i.e., methylated guanine and 2′-deoxyguanosine at position O6) are highlighted in red squares. To illustrate these reactions, guanine and guanosine bases are focused on, but similar products are also obtained for adenine and adenosine with methylation at position N3. These products should not, however, interfere with the analysis of O6-methylguanines or O6-methyl-2′-deoxyguanosine.

In order to assess (i) which target macromolecules (DNA, RNA, others) ultimately define the action of TMZ, (ii) which of their subunits are most relevant to be targeted, and (iii) what fraction of these needs to be methylated, it appears necessary to be able to discriminate and quantify methylated guanines. In the present perspective article, we only focus on workflows for further mass spectrometric measurements (LC-ESI-MS and MALDI-MS). Two pre-analytical approaches appear relevant to assess the molecular SOA of TMZ: (i) the retrieval of deoxyribonucleosides (Figure 4C) and (ii) the retrieval of purines (Figure 4B).

##### Retrieval of Deoxyribonucleosides

The retrieval and MS quantification of nucleosides have previously been described for the determination of decitabine (5-aza-2′-deoxycytidine) incorporated into DNA of treated chronic myelomonocytic leukemia patients [42]. If retrieval of methylated deoxyribonucleosides is performed, the workflows mainly rely on the steps described in Figure 3C, as referenced in Table 2.

DNA digestion can be performed using a cocktail of diverse enzymes (Figure 4A, points 1 to 7). Among the commonly used DNA-metabolizing enzymes are DNases (I [42,52,53] and II), phosphodiesterases (I [42,53,54] and II [54]), nuclease p1 [42,49,52], and alkaline phosphatases [42,52,53,56]. DNases catalyze the hydrolytic cleavage of phosphodiester bonds in DNA backbones [57]. DNase I and II both form two oligonucleotide-end products, 5′-phospho and 3′-hydroxy ends or 5′-hydroxy and 3′-phospho ends, respectively [57]. Phosphodiesterases and nucleases bear the same catalytic functions [42]. Alkaline phosphatases remove 5′-phosphate groups from nucleic acids [58]. Alternatively, commercial nucleoside digestion mixtures composed of exonuclease cocktails can be used to retrieve nucleosides from 5′ ends of single-stranded or double-stranded DNA (e.g., NEB #M0649).

In addition, methyldiazonium can also alkylate RNA [59] and mtDNA (see also Section 2.3), suggesting that they might contribute to the action of TMZ. Regarding RNA, when designing the sample preparation workflow, it is important for the MS analysis and its performance to enable the distinction between methylated deoxynucleosides derived from DNA digestion (Figure 4C) and nucleosides produced from RNA digestion (Figure 4D, see Section 3.3). Depending on these analytical parameters, an RNA removal step may or may not be included in the sample preparation workflow. Because the cellular mtDNA content is negligible compared to the amount of cellular DNA, mtDNA should not influence the measurement of methylated cellular DNA, but this would need to be further investigated.

Microprocessing methods necessitate reducing the number of biochemical steps. However, in order to release coiled DNA from histone complexes, protein digestion might remain a necessary step. Moreover, since DNA enzymes digest single-stranded DNA, there might still be a need for heat-based denaturation.

##### Retrieval of Purines

Methylated purines can be retrieved through DNA depurination methods (Figure 4A–B). During depurination, the nucleobases guanine and adenine are released from the ribose. There are certain conditions under which purine bases dissociate more easily from the ribose (e.g., denaturing conditions such as high temperature, low pH, sublimation, Figure 4A, points 8 and 9) [60,61,62,63,64]. While the depurination rate of single stranded DNA is four times higher than that of double-stranded DNA, this is further accelerated by alkylation, which destabilizes the N-glycosidic bond [60]. Heat-induced hydrolysis of DNA was shown to promote the release of purines and methylated purines such as N7-alkylguanine or O2-alkylcytosine from the DNA backbone [61,63]. Hydrolysis under acidic conditions enhances depurination [61,64]. Detailed methods and workflows using heat-induced and acidic DNA hydrolysis are provided in Table 2. However, it is important to note that heat-induced and acidic depurination of DNA is usually sequence-dependent [61] and might thus bias the downstream interpretation of quantitative results. Indeed, adenosine-rich oligodeoxynucleotides have a half-life eleven-times longer than that of adenosine-cytosine-rich oligodeoxynucleotides under acidic conditions [61]. Sublimation methods also enhance the depurination of DNA but have a limited action on guanines, thus limiting its interest for the study of TMZ action [62].

In addition to depurination by hydrolysis, enzymatic cleavage of purines is a more specific method that may have a lower risk of producing artifacts, but are rarely specific to O6-methylguanines (Figure 4A, points 10 to 12). There are various enzymes targeting specific DNA adducts. For example, the enzyme AlkD specifically excises N7-methylguanine, the most frequently alkylated purine during TMZ treatment [65]. On the other hand, methylpurine glycosylase depurinates DNA from N3-methyladenine and N7-methylguanine [66]. However, these enzymes display no action on O6-methylguanine, largely limiting their utility in the study the antineoplastic activity of TMZ on GBM. Furthermore, endonuclease II was shown to catalyze the release of O6-methylguanine as well as N3-methyladenine from DNA treated with the alkylating agent methyl methanesulfonate [67]. Finally, a DNA enzyme with N-glycosylase activity (10–28 N-glycosylase DNA enzyme) could also be exploited for DNA depurination in the context of the study of TMZ pharmacodynamics [68].

Despite apparent advantages, depurination is mostly conducted in solution with previously purified DNA and with subsequent analysis with LC-MS. It is currently unclear whether enzymatic or hydrolytic approaches would also be suitable for on-surface processing before MALDI-MS or DESI-MS. Furthermore, the previously mentioned tissue loss might present a challenge, as most established depurination protocols involve multiple transfer and washing steps. Additionally, when using a workflow with integrated depurination, RNA removal is a mandatory step to avoid any interference between purines from DNA and RNA (Figure 4B) (see Section 3.3).

Taken together, the technical challenges of depurination methods (the high risk of sample loss with limited possibilities to simplify the workflow; sequence-dependent depurination) mean that depurination may not bring immediate benefit for the specific quantification of O6-methylguanine (in DNA and RNA) using MS.

### 3.3. Analysis

The biological action of TMZ is believed to be triggered by O6-methylguanines (Section 2.3), but guanines are methylated by methyldiazonium at positions O6 and N7, the latter being the preferred site because it is more nucleophilic (Figure 1E). To assess how the biological action of TMZ correlates with its chemical action, it is necessary to distinguish O6-methylguanine quantities from N7-methylguanine ones. To avoid important artifacts in the evaluation of the action of TMZ, all possible sites modified by TMZ metabolites (Figure 1E,F), including the intended molecular SOA (i.e., position O6 on DNA guanines), as well as alternative molecular SOAs (i.e., DNA or RNA adenines, RNA guanines, and N7-methylguanines), should therefore be evaluated using LC-MS/MS, or MALDI-MS(/MS), or both of these analytical methods.

A major source of artifacts in the quantification of TMZ chemical action could be the overlapping signals of N7- and O6-methylguanines, when neither MS/MS fragmentation, nor LC or ion mobility separation is performed. This issue exists for the analysis of nucleosides (Figure 4C, see Section 3.2.2, “Retrieval of Deoxyribonucleosides”) since both N7- and O6-methyl-2′-deoxyguanosine have the same mass, and their expected fragments are, respectively, N7-methylguanine and O6-methylguanine, which also both display the same mass [49]. The issue would also exist using any method that only measures fragments (e.g., using classic MS/MS methods [69] or when in-source decay events (ISD) take place during MALDI experiments) since the fragments of N7- and O6-methyl-2′-deoxyguanosine are N7- and O6-methylguanines, respectively. When considering N7- and O6-methyl-2′-deoxyguanosine for quantification, a separation based on chemical properties other than the mass would then be necessary to permit their discrimination. Here, LC or ion mobility [69] could be considered. When considering their fragments (i.e., N7- and O6-methylguanines) for quantification, only the separation of N7- from O6-methylguanines based on chemical properties in the gas phase would be possible, i.e., using ion mobility [69]. Alternatively, MS3 or pseudo MS3 (in the case of MALDI-ISD) may allow discrimination between fragments from N7- and O6-methylguanines. The issue is the same for the analysis of purines (Figure 4B, see Section 3.2.2, “Retrieval of Purines”) with N7-methylguanine and O6-methylguanine as final reaction products. Separation methods based on chemical properties (LC or ion mobility) or MS/MS fragmentation, rather than the intact mass, are then necessary to discriminate between MS signals from N7- and O6-methylguanines for further specific quantification.

A second potential source of analytical artifacts for the specific quantification of DNA adducts comes from the potential of methyldiazonium to alkylate RNA and other nucleophilic structures. While DNA is composed of 2′-deoxynucleosides, RNA is composed of nucleosides bearing a hydroxyl group at the 2′ position of the ribose moiety. Therefore, methyl-2′-deoxyguanosines display different masses than methylguanosines (17 Da difference). Therefore, any MS method that analyzes the parent mass of methyl-2′-deoxyguanosine with sufficient MS selectivity would suffice to discriminate products from DNA or RNA. However, the issue would remain when using any method that only measures fragments (e.g., when ISD takes place during MALDI experiments or when using an MS/MS method with MS-selection of the parent mass with low-resolution quadrupoles) since the fragments of methyl-2′-deoxyguanosines from DNA and methylguanosines from RNA are both methylguanines. The same is true for the analysis of purines. In this case as well, the products from DNA and RNA are exactly the same—methylguanines—and no separation method would discriminate between products from DNA and RNA. Therefore, when using depurination during sample preparation, RNA removal needs to be performed first (Figure 4A,B).

#### 3.3.1. LC-MS/MS

O6- and N7-methyl-2′-deoxyguanosines can be separated by LC [49]. For the development of LC-MS/MS methods for nucleoside monitoring, it is therefore important to optimize the separation parameters in order to obtain two distinct LC peaks for methyl-2′-deoxyguanosines. The large mass difference of 17 Da between nucleosides and 2′-deoxynucleosides helps with the selection of the 2′-deoxyguanosine parent ion originating from DNA before fragmentation, without interference from RNA products. However, depurination methods inevitably lead to signal interference by guanines and methylated guanines from RNA (Section 3.2.2, “Retrieval of Purines” and Section 3.3). Therefore, RNA removal is necessary, when using any MS method on depurinated samples. However, O6- and N7-methylguanines from DNA obtained after acidic hydrolysis-based depurination can be separated and analyzed by LC-MS/MS [50,51], and distinguished using specific parent-daughter transitions in multiple reaction monitoring (MRM) mode. 

Before LC-MS analysis, solid phase extraction (SPE) can be used for the prepurification of purine bases or nucleosides, as described in the research works referenced in Table 2. Suitable SPE approaches include C18 [70], porous graphitic carbon (PGC) [71,72], phenylboronic acid (PBA) [73,74], hydrophilic lipophilic balances (HLB) [70,75,76,77,78], cation exchange (e.g., MCX) [79], and weak anion exchange (WAX) [80] phases. The comparison of nucleotide purification by (i) polymeric materials with polar and non-polar functionalities (ABN, ENV+, and HLB), (ii) ion-exchange sorbents (anionic (MAX) and cationic (MCX)), and (iii) an affinity sorbent (Affi-gel, to retain compounds with a cis-diol group in their structure) indicated that HLB provided the highest versatility for the purification of the largest panel of nucleotides [75]. Because of the limited sample size, attention must be paid to the available commercial solutions for SPE. In the context of nucleoside purification for evaluating the effect of TMZ on DNA, C18 and HLB remain the most suitable commercial methods for purifying very small sample volumes. Alternatively, technical workarounds can be used to create self-assembled µ-SPE cartridges as already described for HLB cartridges [78].

#### 3.3.2. MALDI-MS

MALDI-MS analysis on tissue sections cannot be coupled to LC separation; thus, in principle, it does not allow for the discrimination between the different methylated forms of 2′-guanosines and guanines. However, MALDI-MS can be coupled to ion mobility (IM) in order to separate ions after ionization. It still has to be determined whether ion mobility can efficiently separate methylation products. In principle, the resolution of the quadrupole in most MALDI-MS instruments should allow the selection of methyl-2′-deoxyguanosine without interference from RNA products. In any case, for the analysis of purines, RNA-derived purines interfere with DNA-derived purines, and therefore RNA removal is also necessary using MALDI-MS methods.

Based on this information, methods that rely on nucleoside analysis appear to open up more flexibility in terms of downstream analytics.

When performing MALDI-MS analyses, the choice of the MALDI matrix is of utmost importance to maximize the chances for detection and quantification of alkylated guanines. Among the candidate matrices for the analysis of nucleosides and purines, classical MALDI matrices for small-compound ionization can be considered, such as 2,5-dihydroxybenzoic acid (DHB) [78,81,82] and α-cyano-4-hydroxycinnamic acid (CHCA) [81,82]. Alternatively, graphene flakes have been proposed for the matrix interference-free analysis of small molecules including guanosines and 2′-deoxyguanosines [83]. 2,3,4,5-tetrakis(3′,4′-dihydroxylphenyl)thiophene (DHPT) was also developed for the analysis of low molecular weight amines and appeared to be efficient for the ionization of guanines in positive mode [84]. In parallel, MALDI matrices reported for the ionization of oligonucleotides might also have to be considered for the ionization of nucleosides and purines, such as 3-hydroxypicolinic acid (3-HPA), 2,4,6-trihydroxyacetophenone (2,4,6-THAP), 2,3,4-trihydroxyacetophenone (2,3,4-THAP), picolinic acid (PA), 3-aminopicolinic acid (3-APA), 6-aza-2-thiothymine (ATT), 5-methoxysalicylic acid (5-MSA), quinaldic acid (QA), pyrazinecarboxylic acid (PCA), 3-hydroxycoumarin (3-HC), and 3,4-diaminobenzophenone (DABP) [85].

MALDI-MS can also induce analytical artifacts from ISD events (Figure 4) when measuring the biochemical action of TMZ. It is noteworthy that for the analysis of oligonucleotides, it was reported that the use of some nucleobase derivatives could induce ISD [86]. Possible ISD events have to be monitored during MALDI analyses of nucleosides and nucleobases.

#### 3.3.3. Quantification Approaches: Development and Validation

Most of the quantification methods used for drug monitoring are based on stable-isotope dilution (SID) methods or on derived methods. SID consists of spiking a stable-isotopically labeled (SIL) analog of the targeted analyte as an internal standard (IS) into (LC-MS/MS) or onto (MALDI-MS) the sample at a known concentration [87]. By normalizing the analyte response with the SIL-IS response and using calibration standard (CAL) and quality control (QC) samples, it is then possible to build calibration curves and account for recovery and biological matrix effects for reliable quantification.

Any quantification assay used to support clinical trials must be validated in accordance with the pertinent regulatory guidelines for bioanalytical method validation (BMV) [7,46,47,48]. Together with the calibration samples used to create calibration curves for the quantification of clinical samples, BMV requires the creation of dedicated QCs containing different concentrations of the analyte to quantify. These QCs can verify that the developed quantification method is specific, accurate, and precise in biological matrices from different individuals and that the extraction methods used are reproducible [7]. Depending on the approach used (e.g., LC-MS/MS or MALDI-MS analysis), QC generation can be very laborious, and BMV can reach different levels of complexity [7].

##### Quantification of Temozolomide

Although we would not expect TMZ to be present in biopsy samples (see Section 3.2.1), the method for TMZ quantification by LC-MS/MS in animals for drug development as performed by Goldwirt and coworkers [45] would be the most straightforward method. Details on the method for quantification and its validation are provided in Table 2. Conversely, the development and validation of desorption/ionization methods for TMZ in tissues would necessitate additional tests for the development of additional QC [7]. For the development of the method, while processing samples in acidic condition to prevent the decay of TMZ (see Section 3.2.1), it would be important to carefully choose the solvent for the solubilization of the standards [88] and to carefully choose the mode of deposition when imaging is used [7] to avoid artifacts during the creation of the calibration curves [7]. For the method validation, the development of dedicated QCs (i.e., “mimetic QCs” or QC MIM—QC samples designed to mimic what should happen in clinical samples) would be necessary to determine the reproducibility of extraction of the drugs using spraying or spotting methods [7].

##### Quantification of Methylated Guanines and Guanosines

Compared to the quantification of free xenobiotics, the difficulty in quantifying methylated guanine residues lies in the fact that (i) the compound of interest is endogenous to the biological matrix and incorporated in a large structure, and that (ii) in the sample processing step, chemical reactions (enzymatic or non-enzymatic hydrolysis) are necessary to retrieve the compounds of interest. These two parameters are important for the choice of quantification method and for the evaluation of important quality parameters in BMV, such as recovery.

Several methods have been developed for the quantification of methylated guanines and 2´-deoxyguanosines by LC-MS/MS (see Section 3.3.1), as detailed in Table 2. It is noteworthy that these methods were performed either from extracted DNA exposed to an alkylating agent or from model samples that can be obtained in relatively large amounts. These only give some hints of the possible quantification approaches that require adaptations in order to perform this quantification in the biological context of the TMZ treatment of GBM.

It is also important to note that in none of these methods, the CALs and QCs were prepared in the biological matrix. CALs and QCs were “free” purine bases or nucleosides (i.e., prepared in buffer or aqueous solvent) and not DNA with known concentrations of purine bases that would be subjected to the enzymatic or non-enzymatic hydrolytic retrieval of nucleosides or purines, as it is usually performed for the quantification of clinical samples. When the use of CALs and QCs, prepared as recommended by the regulatory guidelines, is not possible, it is important to use workaround approaches as detailed in Table 2 to assess important quality parameters, such as isotopic dilution, as used by Hu and co-workers [51], to determine whether the recovery of purines after hydrolytic depurination is close to 100%. If so, it can be assumed that the quantification using “free” purine bases or nucleosides for calibration is exact.

Without calibration standards that fully mimic the samples being processed and quantified, classical approaches to measure recovery cannot assess nucleoside or purine hydrolysis efficiency. For instance, Chilakala and co-workers [42] evaluated sample recovery through possible sample losses during the digestion process but not hydrolysis yields. It is possible that the absolute quantification of a compound hydrolyzed from DNA is biased if hydrolysis yields do not reach 100%.

However, approaches measuring the percentage of methylated nucleosides would be particularly attractive as a means of correcting for artifacts due to incomplete hydrolysis. This approach would, however, necessitate assuming that hydrolysis yields are the same between native and methylated purines. Therefore, considering the abundance of available molecular SOAs (Table 1, P6) would correct possible calibration artifacts and also potential sequence-dependence of hydrolysis.

Alternatively, mimetic calibration standards for the quantification of native and methylated nucleosides or purines would consist of double-strand, coiled, and histone-packed DNA incorporated with a known amount of the native and methylated purines of interest. Although such a calibration standard would reduce bias for calibration, their chemical design appears particularly challenging.

In conclusion, despite possible quantification bias due to possible incomplete hydrolysis of purines or nucleosides, quantification of methylated purines relative to native ones or to total quantities of DNA appears to be the proper method for the evaluation of methylation levels in tissues from TMZ treated patients. This approach would correct for any possible heterogeneous distribution of the cellular and molecular SOAs in the tissue and their exposure to reagents during sample preparation.

In Section 3, the detailed background on the workflows used in previous studies for the retrieval and analysis of methylated purines and nucleosides, together with the particular considerations to be taken in the analysis of human tissues, facilitates a more informed perspective on future possible workflows to follow. The possible low quantities of samples and the heterogeneous proportion of cancer cells in tissues would necessitate using dedicated methods for sampling such as LMD, which allow a detailed correlation with digital histology. Sample preparation may need to be reduced to the minimum number of steps to avoid sample loss. Ideally, only DNA digestion should be retained if this step suffices for the further non-biased quantification of DNA adducts. Although DNA digestion can be performed using selected enzymes, a multienzymatic cocktail may be the most efficient and safest approach. The quantification of methylated 2′-deoxyguanosines, normalized by the quantification of 2′-deoxyguanosines, may represent the most informative approach, by taking into account the unmodified molecular SOA counterpart. LC-MS/MS appears to be the most versatile method, possibly permitting the simultaneous quantification of modified species originating from RNA and DNA with high specificity.

## 4. Biological Models to Study the Action of TMZ at SOAs

Suitable biological models to prove method feasibility need to be designed. In this regard, cellular models would permit initial method development for sample preparation and MS analysis. Investigation in cell cultures would also allow evaluating in vitro the fate of cells under the action of TMZ (i.e., cell arrest, apoptosis, or necroptosis). This would also allow the investigation of the influence of off-target methylations at alternative molecular SOAs on TMZ therapy efficiency. Using cell cultures, the culture medium would also be of interest in the evaluation of the fate of TMZ over time, as well as of alkylated guanines in the extracellular environment, after possible cell death. Three-dimensional cell cultures (organoids, spheroids) of GBM [89,90] and culture-based BBB [91,92] also represent attractive models to study the action of TMZ in the intended cellular and molecular SOA and the permeability of TMZ in the anatomical SOA, respectively. Organoids can be considered the hyphen between regular cell culture and animal tissues, and they do not present ethical issues as animal models do. After culture and drug exposure, organoids can indeed be embedded and processed like a tissue, e.g., with sectioning, staining, digital histological investigation, and LMD. However, it is important to consider that in vitro observations may not be directly extrapolated to in vivo events. For instance, the in vitro degradation kinetics of TMZ at neutral pH appears to be longer than in vivo [93]. On the other hand, animal models (e.g., mice dosed with TMZ followed by tissue collection at regular time points) would represent the most accurate and versatile option for defining the pharmacokinetics of TMZ in the circulation and the anatomical SOA, as well as the chemical modifications of the target of TMZ at its potential molecular SOA. Although it is suggested that TMZ has a cytostatic effect, it would be important to approximate (i) how long the cells bearing the modified guanines remain in the brain (see Section 2.3 and Section 2.4), until cell necroptosis or apoptosis, (ii) whether they keep their methylated DNA, (iii) how long it takes for resistant cells to recover and exchange methylated guanines, and (iv) whether alkylated purines are still present in the normal brain microenvironment after the disappearance of tumor cells under TMZ exposure. Animal models would also give access to tissues to monitor guanine alkylation in alternative anatomical and cellular SOAs for complete ADME studies and correlations with possible beneficial and adverse effects. A validation of the proof-of-concept in human tissues would consist of the comparison between TMZ-naïve and TMZ-treated patients with the same time interval between dose administration and biopsy collection. A realistic maximum time interval would have to be estimated from analytical data retrieved from animal experiments.

## 5. Considerations for the Analysis of Alternative Sites of Action

The action of TMZ may not be restricted to intended SOAs (see Section 2.4). Considering alternative SOAs would permit the most comprehensive investigation of the action of TMZ in GBM. Pharmacological data suggest that both TMZ and MTIC display a T_max_ at 1.5 h [14]. Therefore, although displaying a shorter T_max_ in plasma than its half-life, TMZ decays before reaching the circulation, and may therefore alkylate alternative SOAs in the digestive tract. TMZ may also decay after its distribution in alternative anatomical SOAs and alkylate alternative molecular SOAs in the tissular extracellular matrix and cells. Investigations in humans do not allow for the retrieval of alternative anatomical SOAs as any surgery takes place in brain. However, investigations in animal models do permit the retrieval of alternative anatomical SOAs. Regarding the analysis of methylated RNA, it would be possible to analyze it together with methylated DNA using sample preparation methods allowing the preservation of RNA in the samples and analytical approaches allowing for specific detection of methylated ribonucleosides (see Section 3.2.2). The analysis of alkylation of mtDNA would, however, necessitate dedicated procedures for its extraction from samples [94].

Regarding the intended molecular SOA (DNA), it is known that methylation of the ribose-phosphodiester backbone of DNA also happens under the effect of TMZ. It is also not clear if methylations are restricted to one site of purines or if more than one site can be methylated. It would be useful to explore more deeply possible sites of methylation in all moieties of DNA. This would necessitate the performance of initial discovery experiments before selecting the adduct to quantify or the use of multiplexed methods for the quantification of multiple adducts.

## 6. Measurement of the Biological Action of Temozolomide

Besides the targeted quantification of the chemical action of TMZ at its molecular SOA, further “omics” analyses at the anatomical and cellular SOAs would also be able to provide information about the downstream biological action of TMZ (concentration-response relationship) and about the repair mechanisms opposing its cytotoxic action (dose-concentration relationship).

### 6.1. Genomics and Transcriptomics

The impact of TMZ can be measured using genome-wide molecular approaches, such as genomics and transcriptomics (Figure 1G). Bulk DNA- and RNA-sequencing from primary and recurrent fr/fr glioma tissues has the potential to highlight genes with altered expression in recurrent tumors. Molecular alterations can also be measured using spatial transcriptomics, in which molecular changes in the TMZ-treated recurrent tumors can be analyzed as a function of the spatial characteristics of gliomas. Spatial transcriptomic technologies are becoming more standardized, accessible, and applicable to FFPE tissues, and the comparison of matched primary and recurrent GBMs from archived tissues will be of paramount importance to measure TMZ-associated changes.

The majority of GBM patients receive radiotherapy and TMZ. The treatment course (e.g., the number of TMZ cycles) and the time between end of treatment and relapse or second surgery varies by patient [33]. Therefore, it is difficult to attribute the transcriptional changes in recurrent GBM to TMZ only, especially when the cohort sizes are small.

Ultimately, targeted quantification of chemical action of TMZ could be combined with spatial transcriptomic approaches. Focal areas within tumor tissue harboring a chemical TMZ signature can then be overlaid with molecular changes. Eventually, posttranscriptional events might be altered under the action of TMZ and may also be interesting to investigate (Figure 1G).

### 6.2. Proteomics

As proteins are the final biochemical effectors of the biological action, proteomic analyses represent a considerable asset for the evaluation of the biological effects of TMZ. Proteomic sample processing of small tissue pieces necessitates dedicated consideration for handling in order to avoid sample losses (see Section 3.1). These considerations have recently been termed microproteomics, and approaches for sample processing have been reviewed [44]. On the other hand, approaches based on tissue lysis, protein solubilization, and a modified magnetic bead-based sample cleanup called single-pot solid phase sample preparation (SP3) [95] hold good promises for miniaturization and microproteomic processing.

Besides the miniaturization of sample preparation, a major challenge would consist of elaborating the combined procedures for both DNA hydrolysis and protein digestion. Since protein digestion is necessary to release coiled DNA from histones (see Section 3.2.2, “Retrieval of Deoxyribonucleosides”), this step might be exploited for the integration of nucleoside retrieval within microproteomic protocols. In addition, protein denaturation using tissue incubation at high temperatures is also a recommended step for microproteomic processing of fr/fr [96], which could also be exploited for depurination (see Section 3.2.2, “Retrieval of Purines”).

One role of microproteomics would consist of finding the markers of the biological action and resistance of TMZ, as already suggested [97]. Moreover, proteomics might also provide information about the presence and abundance of drug efflux transporters, which are known to play a role in GBM resistance to TMZ [98] (Table 1, P4).

Since chemical modifications such as methylations can also be measured using proteomic analyses, it would also be possible to screen the panel of methylation on proteins. Direct chemical actions could then be correlated to other biological effects also at a proteomic level. In that regard, histone methylations could also be specifically monitored using dedicated proteomic approaches [99] in order to correlate these modifications with epigenetic events [32]. The eventual biochemical effects of TMZ may be posttranslational modifications (PTM) on proteins and PTM crosstalks, which may also be monitored using dedicated proteomic assays [100] (Figure 1G).

## 7. Applications

Although great progress has been made in understanding the chemical and biological effects of TMZ, they are not yet fully understood. The importance of monitoring TMZ would be two-fold, (i) for drug development, to better control possible events when new drugs (including targeted therapies) are tested in clinical trials in combination with TMZ and (ii) for precision medicine, to control whether TMZ actually reaches SOA in sufficient amounts and exerts the expected molecular (transcriptomic, proteomic) effect. Known resistances to TMZ treatment include pre-transcriptional events (e.g., functional MGMT and dysfunctional MMR machineries), post-transcriptional events (e.g., microRNA [98]), and post-translational events (e.g., histone modification) [5,97] (Table 1, P8); however, they also involve the tissue microenvironment (such as hypoxia, gap junctions, glioma stem cells) and the action of drug efflux transporters (Table 1, P8) [98]. Combining methylation efficiency on DNA and alternative molecular SOAs with functional parameters could lead to new ways of increasing tolerability and improving dosing schedules and possibly even routes of administration. It could also help in establishing biomarkers of TMZ response that are more easily available than brain tissue biopsies.

The quantification of alkylated guanines or guanosines (especially in the O6 position) would allow for a better monitoring of the chemical action of TMZ in TMZ-resistant patients and patients in clinical trials testing combinations of TMZ with new drugs. In resistance studies, the timing of tissue collection after treatment is a critical parameter that must be considered to assess whether alkylated guanines will still be detectable. The probability of finding living cells in tissue after TMZ treatment may be limited because a second surgery in an eligible patient occurs months after the last TMZ dose (see Section 2.4). In the absence of living cells bearing the TMZ-modified guanines, the comparison of remaining tumor cells from TMZ-responders and TMZ-resistant patients may not display any difference. However, since previous studies hint that TMZ effects might be cytostatic rather than cytotoxic (see Section 2.3 [26,27]), arrested cells might still be present in the tumor at the time of tissue collection. The quantification of alkylated guanines holds greater promise for monitoring TMZ in the context of clinical trials of new drugs. Since time points for biopsy collection are closer to the time of treatment, the probability of finding cells bearing the modified molecular SOA are higher. Finally, the monitoring of TMZ at its SOAs would find its most straightforward application during drug development in animal models. In this context, time points for tissue collection can be finely monitored for pharmacokinetics.

In parallel, omics analysis (transcriptomics, proteomics) would allow for the broadest monitoring of the side chemical actions and induced biological actions (Figure 1H). In resistant patients, the altered abundance of markers of the biological effects of TMZ would confirm a reduced TMZ effect. Additional markers of resistance would provide information about the mechanism of the appearance of resistance. In patients selected for clinical trials of new drugs together with the standards of care, markers of effect and/or toxicity of TMZ could be monitored in order to distinguish the effect of the new compound from the effect of TMZ. As with the quantification of alkylated guanines, the level of significance of performing omics analyses would depend on the probability of finding living cells exposed to TMZ, or resistant, apoptotic, and necroptotic cells. Theoretically, omics analyses may hold more promises for molecular pharmacodynamics in the context of clinical trials and drug development in animal models.

Altogether, the combination of the absolute quantification of the chemical action of TMZ and omics analyses represent an extended package for molecular drug monitoring.

## 8. Conclusions

Approaching the SOAs of drugs in clinical pharmacological investigations would have a great impact in precision medicine, during clinical trials, and for patient monitoring. The absolute quantification of the chemical action of TMZ in GBM represents a challenge. Given the high reactivity of its carbocation intermediate with a large panel of biomolecules, the quantification of adducts at intended molecular SOAs might not suffice to explain the complete biological action of TMZ. The clinical intended effects and side effects of TMZ may actually be related to its chemical action on DNA, as well as on RNA, mtDNA, and proteins at multiple anatomical sites. Covering all these chemical and biochemical events would rely on multiplexed quantification methods and multi-omics approaches. The small size of sample available also represents an important analytical challenge regarding drug action quantification and omics investigations. However, recent investigations and developments indicate that these tasks can be addressed. Addressing these challenges would be rewarded by a considerable impact on the clinical pharmacology of TMZ and new GBM drugs administered in combination with TMZ, including targeted therapies.

## Figures and Tables

**Table 1:** Key parameters to evaluate when studying drug pharmacokinetics at the sites of action (SOAs) [6]. Param.: Parameter.

Param.	Description
P1	Dose of therapeutic compound
P2	Unbound drug concentration in plasma (drug free fraction)
P3	Localization of the drug’s cellular SOA (e.g., intravascular or interstitial compartment)
P4	Expression and activity of transporters at the cellular SOA
P5	Unbound and bound drug concentration on or in the cellular SOA
P6	Abundance of the molecular SOA and number of available specific binding sites
P7	Chemical interaction between the drug and its molecular SOA
P8	Alternative (off-)site binding
P9	Concentration of drug metabolites in the cellular SOA
P10	Expression and function of drug metabolizing enzymes and other clearance mechanisms

**Table 2:** Referenced workflows for analysis/quantification of temozolomide and DNA/RNA adducts.

Sample Type	Targeted Compound	Sample Preparation	Enzymatic Digestion (Specificity Indication)	Analytical Method	Quantification Strategy	Quality Parameters	Ref.
Tissue (mice dosed with TMZ)	TMZ	Low pH conditions/Tissue homogeneization/Protein precipitation	n.a.	LC-MS/MS 4 min LC gradient MRM (QqQ)	Tissue homogenate spiked with TMZ and theophylline (IS) for CALs and QCs	Partial validation (assay precision, accuracy, recovery, linearity, specificty, and matrix effect) [7,46,47,48]	[45]
Cells (cell lines and patient cells exposed with decitabine)	Decitabine (5-aza-2′-deoxycyti dine) incorporated in DNA	Cell lysis/Protein digestion (proteinase K)/DNA extraction/RNA removal (RNase)/DNA denaturing and hydrolysis/IS spiking/Deproteination/ Evaporation/Reconstitu tion	DNA hydrolysis: DNase I (DNA phosphodiester bonds) NPI (RNA and ssDNA phosphodiester bonds) PDE I (phosphodiester bonds) ALP (removal of 5′-phosphate)	LC-MS/MS MRM (Q-Trap)	BIS-TRIS buffer spiked with deox ycytidine (dC), deoxyguanosine (dG), 5-methyl-2′-deoxycytidine (5 mdC) for CALs and QCs [DNA] evaluation: [DNA] (mg/L) = [dG] × 618 (g/mol)/0.41 Calculation of [decitabine] in pmol/µg of DNA DNA methylation evaluation: % methylation = [5 mdC]/([dC] + [5 mdC]) × 100%	Partial validation (assay precision, accuracy, recovery, linearity, and matrix effect) [7,46,47,48]	[42]
DNA (from calf thymus) Liver tissues (from rat exposed to MNU)	N7- and O6-methyl-2′-deoxy guanosine	DNA: DNA methylation by MNU in buffer/DNA precipitation. Tissues: Homogeneization and cell lysis/RNA digestion/Protein digestion (protease K)/DNA precipitation. All samples: DNA hydrolysis/ultrafiltration (<30,000 g/mol)	All samples (DNA hydrolysis): NP1 ALP	LC-UV-MS/MS 23-min gradient MRM (QqQ)	LC mobile phase spiked with N7- and O6-methyl-2′-deoxyguanosines, and [^2^H_3_]-N7- and [^2^H_3_]-O6-methyl-2′-deoxyguanosines (IS) for CALs	Stability experiments at −20 °C, room temperature, and 37 °C using N7- and O6-methyl-2′-deoxyguanosine solutions in Tris buffer. Recovery: DNA hydrolysates spiked with standards at different concentrations before and after ultrafiltration compared to standards in water without processing.	[49]
DNA (from salmon testis) exposed to MNU and MMS	N7- and O6-methylguanines	DNA methylation by MNU or MMS in buffer/DNA precipitation and isolation/DNA depurination by simultaneous heat-induced hydrolysis (90% FA, 85 °C for 60 min)	n.a.	LC-MS/MS 8-min gradient MRM (QqQ)	Water with 5% FA spiked with N7- and O6-methylguanines, and [^2^H_3_]-N7- and [^2^H_3_]-O6-methylguanine (IS) for CALs and QCs.	Partial validation (assay precision, accuracy, linearity) [7,46,47,48] Stability experiments: Freeze-and-thaw and 45-day stabilities at −20 °C. Recovery: control DNA acidic hydrolytes spiked with QC concentrations of N7- and O6-methylguanines and reated IS, compared to QC samples (standards and IS spiked in water with 5% FA).	[50]
DNA (from calf thymus) exposed to MMS	N7- and O6-methylguanines, and N3-methyladenines	DNA methylation by MMS in buffer/DNA precipitation/DNA depurination by simultaneous heat-induced and acidic hydrolysis (0.1 M HCl, 80 °C for 30 min)	n.a.	LC-MS/MS 4-min gradient MRM (QqQ)	Water/MeOH/TFA 97:3:0.1 (*v*/*v*/*v*) spiked with N7- and O6-methylguanines, and N3-methyladenine, and ^15^N_5_-N7-methylguanine (IS), d_3_-O6-methylguanine (IS), and d_3_- N3- methyladenine (IS) for CALs and QCs.	Partial validation (assay accuracy and linearity) [7,46,47,48] Intra- and inter-day precision calculated between replicates of untreated DNA spiked with unlabeled standard mixture of fixed concentrations. Recovery: untreated DNA spiked with unlabeled standard mixtures of three different concentrations. Matrix effect: comparison of IS areas between CALs and DNA samples.	[51]
DNA (from calf thymus) exposed to cisplatin	1,2 guanine-guanine intrastrand cisplatin adducts (CP-d(GpG))	DNA hydrolysis/SPE (SCX and C18) or HPLC clean-up	DNAase I NP1 ALP	LC-MS/MS 22-min gradient SRM (QqQ)	Preparation of a CP-d(GpG) analyte standard in 10 mM ammonium acetate and of a ^15^N_10_-CP-d(GpG) IS in 10 mM ammonium acetate with 0.1% glacial acetic acid for CALs.	Linearity: one calibration curve. Interday- and interpreparation-precisions: four replicates of CP-d(GpG) at different concentrations. Recovery: analyte standard processed with or without clean-up method.	[52]
DNA (from human placenta) exposed to FA	Hydroxymethyl deoxynuclosides	DNA incubation with FA/DNA precipitation/DNA hydrolysis	DNAse I PDE ALP	LC-UV	Creation of standard hydroxymethyldeoxydeoxynuclosides by exposition of deoxynucleosides with FA. Calibration curves built without IS normalization.	n.a	[53]
Oligonucleotides (synthetic) exposed to cisplatin	Guanine-guanine (GG), guanine-adenine (GA), and adenine-guanine (AG) adducts with cisplatin adducts (cis-Pt(NH_3_)_2_)	Oligonucleotide incubation with cysplatin/Separation of unreacted and cisplatin derived oligonucleotides/DNA hydrolysis	PDE I or PDE II	LC-UV	n.a.	n.a.	[54]
Urine (from human)	O6-carboxymethyl guanine, O6-carboxymethyl-2′-deoxyguanosine, O6-methylguanine and O6-methyl-2′-deoxy guanosine	DNA depurination by simultaneous heat-induced and acidic hydrolysis (0.1 M FA, 70 °C for 1 h)/SPE (C18) clean-up	n.a.	LC-MS/MS 23-min gradient SRM (QqQ)	Synthetic urine spiked with serial dilutions of O6-carboxymethylgua nine, O6-carboxymethyl-2′-deoxyguano sine, O6-methylguanine (control) and O6-methyl-2′-deoxyguanosine (control) and fixed concentrations of tubercidin (IS) for CALs and QCs.	Partial validation (assay linearity, intra-and inter-day accuracy and precision) [7,46,47,48]	[55]

ALP: alkaline phosphatase; CAL: calibrator; FA: formic acid; HPLC: high-performance liquid chromatography; IS: internal standard; LC: liquid chromatography; MMS: methyl methanesulfonate; MNU: methylnitrosourea; MRM: multiple reaction monitoring; MS: mass spectrometry; MS/MS: tandem MS; n.a.: not applicable; NP 1: nuclease P1; PDE: phosphodiesterase; QC: quality control; QqQ: triple quadrupole analyzer; QTrap: quadrupole-ion trap analyzer; SCX: strong cation exchange; SDS: sodium dodecyl sulfate; SPE: solid phase extraction; SRM: selected reaction monitoring; TFA: trifluoroacetic acid; TMZ: temozolomide; UV: ultra-violet.

## Data Availability

Not applicable.
